# From black-box prediction to transparent insight: the status quo and paradigm shift of explainable artificial intelligence in hepatocellular carcinoma research

**DOI:** 10.1186/s43046-026-00397-0

**Published:** 2026-08-11

**Authors:** Yuehua Li, Pengfei Li

**Affiliations:** 1 College of Computer Science and Technology (College of Data Science), Taiyuan University of Technology, Taiyuan, China; 2 Shanxi Medical University, Taiyuan, China

**Keywords:** Hepatocellular carcinoma, Explainable artificial intelligence, Medical imaging diagnosis, Biomarker discovery, Prognostic assessment

## Abstract

Hepatocellular carcinoma (HCC) is a malignancy with high global incidence and mortality, whose significant heterogeneity and poor prognosis pose severe clinical challenges. While artificial intelligence (AI) shows potential in HCC imaging, pathology, and prognosis, its “black-box” nature limits clinical adoption. Explainable AI (XAI) aims to reveal the decision-making logic of AI models. This narrative review synthesizes recent advances of XAI across four key domains of HCC research. In imaging diagnosis, techniques such as Grad-CAM and SHAP have enabled semantic alignment between AI outputs and clinical standards like LI-RADS, enhancing interpretability. In biomarker discovery, XAI has progressed from identifying single markers to revealing functional gene modules and molecular subtypes through multi-omics integration. In treatment efficacy prediction, XAI-based models have quantified feature contributions to therapeutic responses, supporting individualized treatment stratification. In prognosis assessment, XAI has enabled dynamic risk stratification by integrating clinical, imaging, and pathological features. However, three cross-cutting limitations persist across these domains: explanations remain predominantly correlational rather than causal, a semantic gap exists between pixel-level heatmaps and high-level clinical reasoning, and most models are static, unable to adapt to evolving clinical data. Current research is moving toward causal inference frameworks, concept-driven interpretability, and interactive, dynamic systems. In summary, XAI is transitioning from a retrospective explanation tool toward a prospective clinical decision partner, yet bridging the gap between explanation and actionable decision support remains the central challenge.

## Introduction

Hepatocellular carcinoma (HCC) ranks among the malignancies with the highest global incidence and mortality rates. Its significant heterogeneity and poor prognosis pose continuous challenges for clinical diagnosis and treatment [1]. Currently, HCC diagnosis and treatment decisions rely primarily on physicians’ clinical experience, while traditional biomarkers like alpha-fetoprotein (AFP) have certain limitations in sensitivity and specificity [2]. In recent years, the application of artificial intelligence (AI) technology in the medical field has brought new opportunities for HCC research. Deep learning technologies have demonstrated remarkable potential in HCC imaging analysis, pathological diagnosis, genomic research, and prognosis prediction [3, 4]. However, most high-performance AI models exhibit a “black-box” nature, characterized by opaque decision-making processes. This lack of transparency makes it difficult for clinicians to understand and trust the model outputs, which consequently limits the promotion and application of AI technology in clinical practice [5]. This opacity poses a particular challenge in HCC, a cancer characterized by substantial inter-tumor and intra-tumor heterogeneity at the molecular, pathological, and imaging levels. A single black-box prediction cannot reveal whether its decision logic is driven by features generalizable across all patients or by features relevant only to a specific subgroup. XAI, by making the reasoning behind each prediction transparent, can help clinicians assess whether a model’s output is trustworthy for a given individual patient, thereby addressing the specific demands imposed by HCC heterogeneity. The development of explainable artificial intelligence (XAI) offers a potential solution to this challenge. XAI reveals the decision-making basis of AI models, enabling clinicians to understand and evaluate the outputs of high-performance AI models in clinical applications [6]. Current major XAI techniques include post-hoc interpretation methods —methods that explain a trained model’s predictions without modifying the model itself—such as SHAP, LIME, and Grad-CAM, and intrinsically interpretable models (such as attention mechanisms and custom interpretable loss functions). These techniques enhance model interpretability in different ways, promoting the application of AI technology in the medical field [7, 8]. This article provides a comprehensive narrative review of the progress, challenges, and future trajectories of Explainable AI (XAI) in hepatocellular carcinoma (HCC) research. Our aim is not to perform a quantitative systematic evaluation of a single intervention, but to integrate and critically appraise the expanding body of work across the key domains of imaging diagnosis, biomarker discovery, therapeutic efficacy prediction, and prognosis assessment. We first synthesize how XAI is currently translating “black-box” predictions into transparent insights across these clinical areas. Subsequently, we analyze the fundamental limitations of the prevailing post-hoc explanation paradigm. Finally, we present a forward-looking perspective on next-generation technical pathways and the necessary paradigm shift towards causal, interactive, and dynamic XAI systems that can evolve into clinical decision partners. Through this structure, we summarize current progress and outline future research directions for advancing XAI towards greater clinical utility and trustworthiness in HCC precision medicine.

## A synthetic overview of the research status: how XAI drives HCC research from black-box prediction to transparent insight

**Fig. 1:**
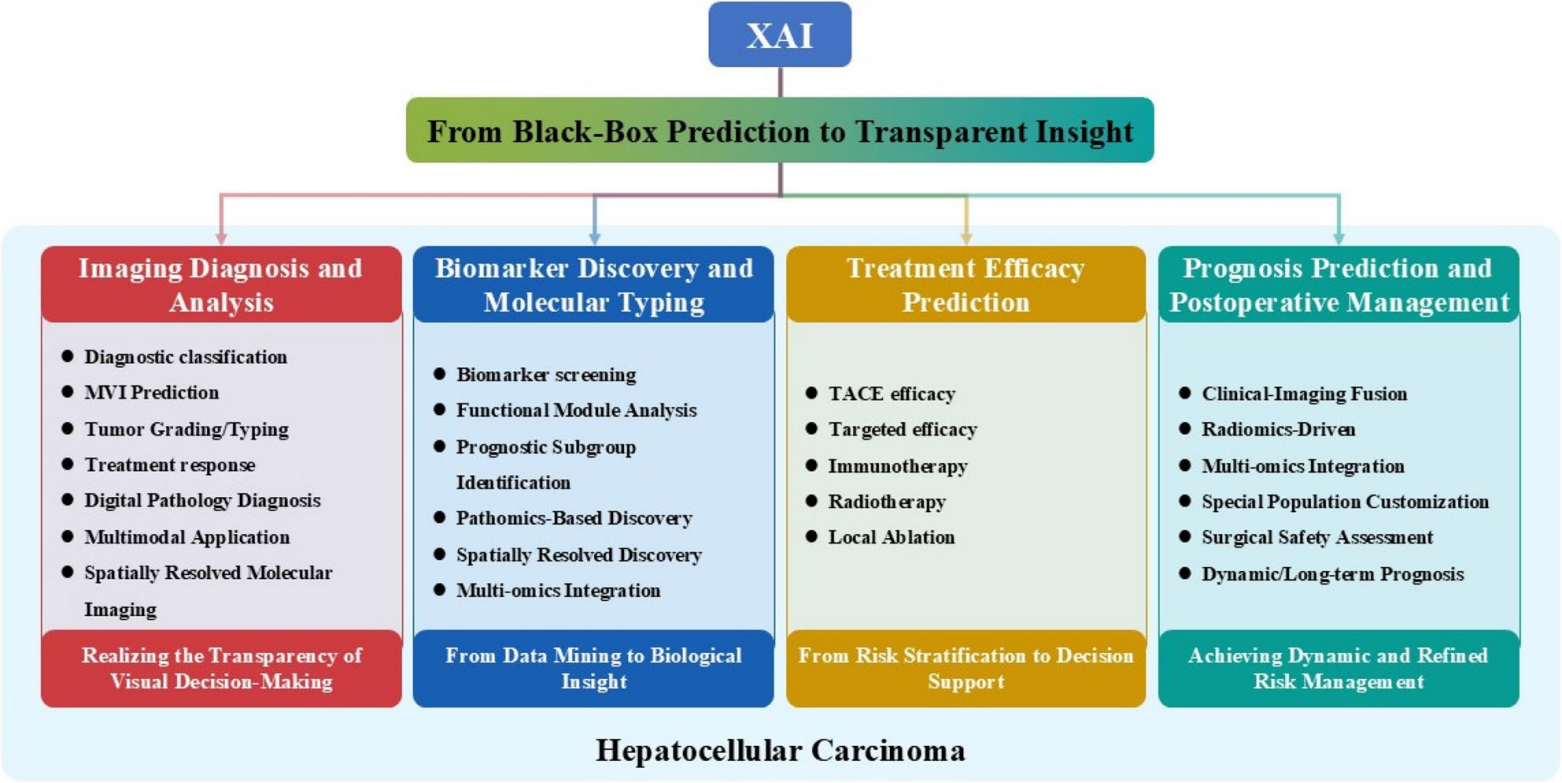
From black-box prediction to transparent insight: an overview of explainable artificial intelligence (XAI) applications in hepatocellular carcinoma (HCC) research. The figure presents a top-down hierarchical framework organized into four core domains—imaging diagnosis and analysis, biomarker discovery and molecular typing, treatment efficacy prediction, and prognosis prediction and postoperative management—each subdivided into major research directions within that area, and summarizes the overarching transformation goal of each domain, collectively illustrating the shift from black-box predictions to clinically interpretable and actionable insights

The core paradigm of current research is to use XAI technology to interpret the decision-making process of high-performance but difficult-to-understand “black-box” machine/deep learning models, making their output transparent and credible. Currently, XAI has been applied across all key areas of HCC research (Fig. 1).

### Application of XAI in HCC imaging diagnosis and analysis: realizing the transparency of visual decision-making

In the field of medical imaging, the core role of XAI is to visualize the “visual focus” and “decision-making basis” of the AI model, aligning and mutually verifying them with the professional knowledge of radiologists (Table 1).

**Table 1:** Summary of representative studies on XAI in HCC imaging diagnosis and analysis

Application Direction	XAI Technology	Core Contribution	Clinical Value	References
Diagnostic Classification	Post-hoc Interpreter, Decision Tree Rules	Achieve LI-RADS feature alignment	Improve diagnostic consistency and interpretability	[9–11]
MVI Prediction	Grad-CAM, Radiomics + SHAP	Discover the importance of the peritumoral area, visualize key regions	Preoperative non-invasive assessment, surgical planning guidance	[12–16]
Tumor Grading/Typing	Multi-task Learning, Feature Sharing	Associate imaging and pathological features, identify cholangiocytic phenotype	Non-invasive grading assessment, molecular subtype identification	[17]
Treatment Response	Longitudinal SHAP Analysis, Habitat Model	Quantify feature contribution, distinguish sensitive/resistant areas	Early efficacy prediction, beyond RECIST standards	[18, 19]
Digital Pathology Diagnosis	Attention Maps, Grad-CAM, Quantification-based XAI (Q-XAI)	Visualizes diagnostic-critical histologic features; bridges AI reasoning with pathological semantics	Aids transparent diagnosis (grading, subtyping); provides computational second opinion	[20–22]
Multimodal Application	Cross-modal Interpretable Analysis	Expand applications to ultrasound, special MRI sequences	Technical universality verification, basis for multimodal fusion	[23–25]
Spatially Resolved Molecular Imaging	Analysis of Molecular Maps, Multimodal Fusion	Decodes macro-imaging phenotypes via underlying spatial molecular patterns.	Provides multi-scale, biologically-grounded explanations; bridges radiologic, histologic, and molecular data layers.	[26, 27]

In diagnostic classification and differential diagnosis, the research focus is on achieving semantic alignment between AI judgment and clinical standards. For example, the post-hoc interpreter developed by Li et al. [9] can decode the activation patterns of deep learning models into LI-RADS features (such as “non-rim arterial phase hyperenhancement”). Jiang et al. [10] optimized LI-RADS classification by extracting random forest feature importance and decision tree rules, generating understandable diagnostic rules. The application of Kim et al. [11] in hepatobiliary phase MRI further expanded the value of XAI in different imaging sequences. Collectively, these studies suggest that XAI can significantly improve the interpretability and acceptability of diagnostic results by transforming model output into semantic concepts familiar to clinicians.

In the field of non-invasive prediction of pathological features, XAI technology effectively reveals the relationship between imaging features and microscopic pathological changes. For example, Liu et al. [12] and Guo et al. [13] used Grad-CAM to confirm that the tumor edge and peritumoral area are key to predicting microvascular invasion (MVI). Famularo et al. [14] and Wang et al. [15] verified this discovery through radiomics combined with SHAP. Fu et al. [16] and Zou et al. [28] further discovered the synergistic effect of intratumoral and peritumoral features. In tumor grading, the multi-task learning framework of You et al. [17] implicitly associated imaging performance with pathological grading. These studies indicate that XAI can extract visual evidence highly related to microscopic pathological changes from images, providing new ideas for non-invasive pathological prediction.

In treatment response evaluation, XAI provides deep efficacy insights beyond conventional standards. For example, Xu et al. [18] used the SHAP analysis of a longitudinal CT radiomics model to quantify the individual contribution of imaging features to the response of combined immunotherapy. The CT habitat model of Shen et al. [19] realized the spatial positioning of efficacy-sensitive and resistant areas through XAI, providing a new perspective for precise treatment. These results highlight the potential of XAI in quantifying efficacy biomarkers and guiding individualized treatment strategies.

In terms of special sequences and modalities, XAI technology also proves its universality. For example, the application of Du et al. [23] in the diagnosis of small hepatocellular carcinoma by ultrasound, and the exploration of Liu et al. [24] and Chen et al. [25] in special MRI sequences, all provide interpretable tools for multimodal image analysis. These studies demonstrate that XAI has been shown to adapt to the characteristics of different imaging techniques and provide a preliminary reliable interpretability foundation for multimodal data fusion.

In parallel to radiological imaging, digital pathology has emerged as a foundational “imaging” modality for HCC. The application of XAI to whole-slide images (WSIs) seeks to render the diagnostic reasoning of AI models for tasks such as tumor grading, histological subtyping, and differential diagnosis transparent and auditable. Techniques like attention-based visualizations and gradient-weighted class activation mapping (Grad-CAM) can highlight histologically critical regions—such as foci of nuclear atypia, architectural distortion, or specific stromal reactions—that underpin model predictions. This bridges the AI’s decision logic with the morphological assessment framework of pathologists, fostering trust and enabling a form of computational second opinion [20, 21].

Furthermore, the frontier of “imaging” is being redefined by spatially resolved omics (e.g., spatial transcriptomics), which generates high-resolution maps of molecular expression registered to tissue architecture—essentially creating “molecular images” of the tumor microenvironment. This data form not only necessitates computational image analysis [26] but also expands the explanatory scope of XAI to include the interpretation of spatial molecular domains. The integrative potential of this paradigm is exemplified in HCC, where spatial transcriptomics has been leveraged to decode the biological basis of specific MRI phenotypes, directly linking macroscopic imaging features to microscopic molecular and cellular spatial patterns [27]. This convergence marks the evolution of imaging-based XAI towards a multi-scale framework that encompasses radiologic, histopathologic, and molecular spatial data layers.

Although XAI has made significant progress in HCC imaging diagnosis and analysis, it still faces some challenges. Most existing XAI methods (such as saliency maps) provide pixel-level explanations, which creates a gap with the diagnostic thinking mode of radiologists based on high-level semantic concepts (such as “washout pattern”). In addition, models sometimes learn data biases unrelated to the essence of the disease (i.e., “shortcut learning”), which may cause the provided explanations not to truly reflect the model’s decision-making logic and may even cause misleading [22, 29].

### Application of XAI in HCC biomarker discovery and molecular typing: from data mining to biological insight

**Fig. 2:**
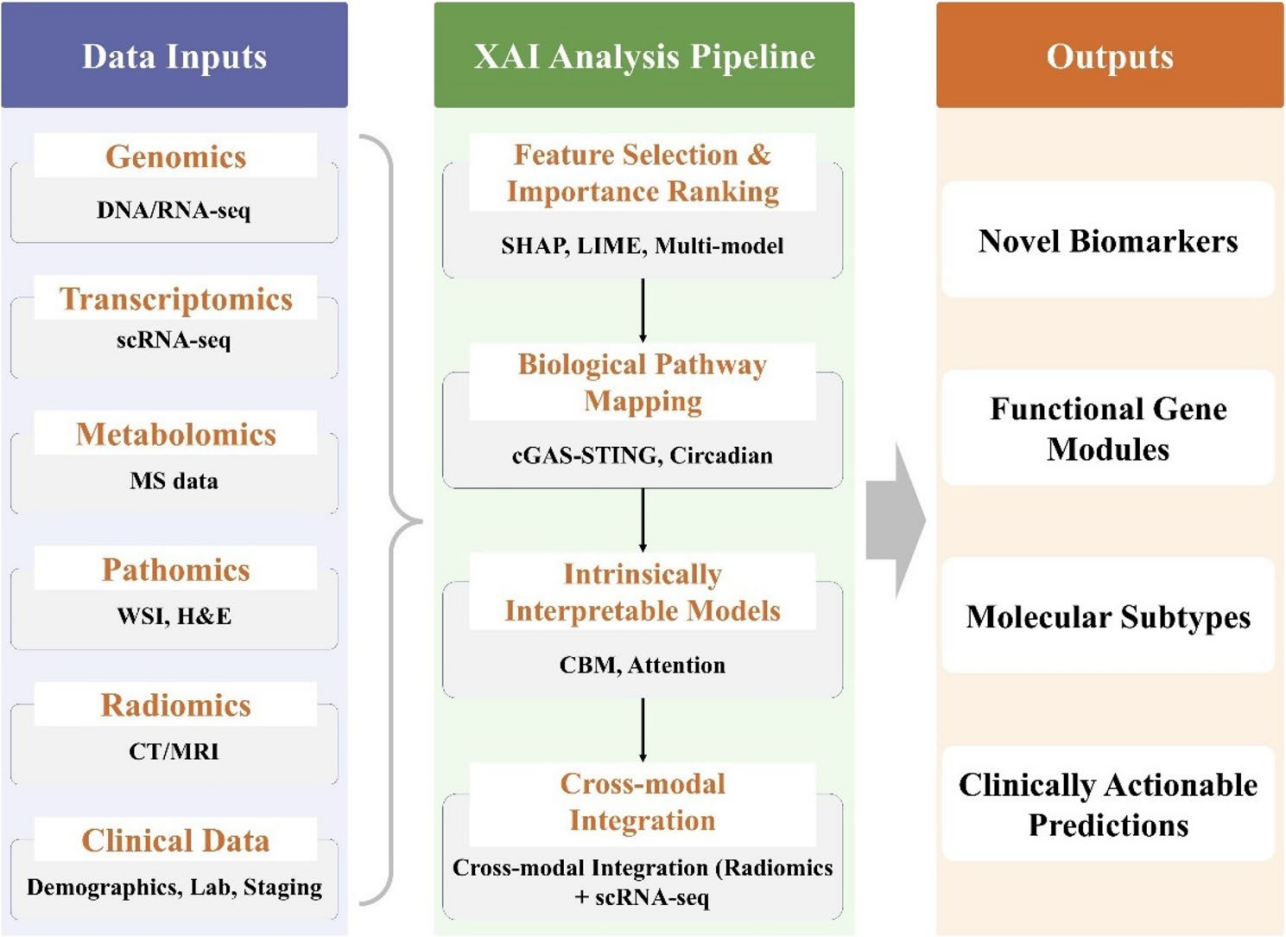
XAI-driven multi-omics integration pipeline for biomarker discovery and molecular subtyping in hepatocellular carcinoma. The pipeline encompasses three stages. Left panel: Data Inputs, including genomics (DNA/RNA-seq), transcriptomics (scRNA-seq), metabolomics (MS data), pathomics (WSI, H&E), radiomics (CT/MRI), and clinical data (demographics, lab tests, staging). Middle panel: XAI Analysis Pipeline, comprising feature selection and importance ranking (e.g., SHAP, LIME, multi-model frameworks), biological pathway mapping (e.g., cGAS-STING pathway, circadian rhythm pathways), intrinsically interpretable models (e.g., concept bottleneck models [CBM], attention mechanisms; CBMs are discussed in detail in sect. "From “pixel heatmaps” to “concept-driven”: bridging the semantic gap in image analysis"), and cross-modal integration (e.g., radiomics combined with single-cell transcriptomics). Right panel: Outputs, including novel biomarkers, functional gene modules, molecular subtypes, and clinically actionable predictions for treatment response and prognosis. Solid arrows indicate the analytical flow from data inputs through XAI-based interpretation to clinical translation

In the field of omics data—here referring to multi-omics, i.e., the integrated analysis of data from multiple molecular layers such as genomics, transcriptomics, metabolomics, together with imaging and clinical phenotypes—the core value of XAI is to act as a powerful “high-dimensional data miner” and “biological hypothesis generator”. Its research level has gradually deepened from single biomarker screening to systematic biological function interpretation and clinical transformation of multi-omics integration. A complete workflow of XAI-driven multi-omics analysis, from data inputs to clinical outputs, is presented in Fig. 2 at the end of sect. "A synthetic overview of the research status: how XAI drives HCC research from black-box prediction to transparent insight" (Table 2).

**Table 2:** Summary of representative studies on XAI in HCC biomarker discovery

Research Level	Representative Methods	Biological Significance	Main Breakthrough	References
Biomarker Screening	Multi-model Verification, AutoML+SHAP	Improve discovery robustness, efficient screening	New gene and metabolite biomarkers	[30–32]
Functional Module Analysis	Network Analysis + XAI	System-level functional interpretation, discovery of gene communities	Insight upgrade from “gene” to “module”	[33]
Prognostic Subgroup Identification	Intrinsically Interpretable Framework	Biological homogeneity typing, driver feature identification	Directly output interpretable subgroups	[34, 35]
Pathomics-Based Discovery	Multimodal Fusion, Weakly-supervised DL, Q-XAI	Links morphological phenotypes to molecular subtypes and clinical outcomes.	Enables non-invasive prediction of MVI and molecular traits from H&E slides.	[22, 36–39]
Spatially Resolved Discovery	Spatial transcriptomics, Multimodal fusion	Links gene expression to tissue architecture; reveals TME spatial heterogeneity.	Identifies spatially informed biomarkers; elucidates niche-specific interactions.	[40–42]
Multi-omics Integration	Cross-modal Association Analysis	Connect imaging, genome, transcriptome data	Provide biological explanation for prognostic differences	[43, 44]

Multi-omics biomarker screening research has established a robust discovery pipeline. Gutierrez-Chakraborty et al. [30] used a multi-model XAI framework to improve the robustness of gene biomarker discovery. Yagin et al. [31] combined AutoML with TreeSHAP to achieve efficient discovery of metabolomics biomarkers. Zhang and Liu [32] used a variety of feature selection methods and cross-validation to ensure the reliability of gene features. These methods together reflect the methodological progress of XAI in efficiently and accurately screening potential biomarkers from massive data.

Systems biology and functional module interpretation are deeper-level applications. Lacalamita et al. [33] analyzed functional modules from the perspective of “gene communities”, elevating the interpretation level from a single gene to the system level. Owens et al. [34] designed an intrinsically interpretable deep learning framework that directly outputs prognostic subgroups with biological homogeneity. Wang et al. [35] combined SHAP with cGAS-STING pathway analysis to explain prediction results from a mechanistic level. These studies show that XAI can reveal complex gene interaction networks and biological pathways, providing deeper biological insights.

Multi-omics integration and clinical translation are the frontier directions. Gao et al. [43] integrated single-cell transcriptomics and radiomics, discovering the association between imaging features and the immune microenvironment. Xu et al. [44] focused on circadian rhythm markers, providing a basis for chronotherapy. These studies establish connections between macroscopic phenotypes and microscopic mechanisms. These related studies demonstrate the frontier exploration of XAI in integrating multi-dimensional data and proposing verifiable clinical hypotheses.

Concurrently, pathomics—the high-throughput extraction of quantitative morphological features from hematoxylin and eosin (H&E)-stained whole-slide images—has become a rich source of directly phenotypic data for biomarker discovery [21, 45]. XAI plays an important role in deciphering the associations between these complex, high-dimensional morphological patterns and clinical outcomes. For instance, interpretable deep learning models can predict microvascular invasion (MVI) status from WSIs, with techniques like Grad-CAM visualizing histologically plausible regions (e.g., macrotrabecular structures) linked to aggressiveness [36]. Moving beyond single-modal analysis, multimodal interpretable frameworks are being developed to fuse pathological image data with molecular profiles. Token-guided fusion mechanisms, for example, can concurrently leverage WSI-derived morphological information, quantitative tumor-stroma ratio, and gene expression data to predict molecular subtypes, while explicitly attributing predictive contributions to each data modality [37]. Similarly, dual-branch exploratory architectures (e.g., DuXplore) separately model and explain the prognostic impact of tissue architecture versus cellular morphology, offering granular, biologically meaningful insights [38]. To further align model explanations with pathological practice, quantification-based XAI (Q-XAI) methods map deep learning features to quantifiable nuclear morphometric features (e.g., size, pleomorphism), directly grounding the explanation in the quantitative diagnostic criteria familiar to pathologists [22]. These approaches collectively advance the field from identifying statistical associations to extracting interpretable histological phenotypes with clear biological and clinical relevance.

Beyond bulk omics, spatial transcriptomics maps genome-wide gene expression within intact tissue architecture, directly linking morphology to molecular phenotype and revealing the spatial heterogeneity of the HCC tumor microenvironment [40]. This technology is pivotal for understanding the spatial determinants of therapy response and resistance [41], and for deconvolving the complex immune ecosystem in specific HCC subtypes [42]. The analysis of such high-dimensional, spatially resolved data presents a major challenge for advanced computational methods. Explainable AI (XAI) is particularly suited to decode these complex patterns, as it can identify spatially informed biomarkers by pinpointing molecular signatures in specific histological regions that predict clinical outcomes.

Although the application of XAI in HCC biomarker discovery and molecular typing has made significant progress, an important link needs to be improved in these studies: most of the associations revealed by current XAI are still statistical correlations. The important biomarkers discovered must undergo rigorous biological experimental verification to finally establish their causality, and this step is still generally insufficient in current research [46, 47].

### Application of XAI in HCC treatment efficacy prediction: from risk stratification to decision support

In the treatment decision-making of HCC, the core value of XAI is to reveal why different treatment options (such as TACE, targeted therapy, immunotherapy) produce differential efficacy in individual patients, thereby providing deep-level decision support for clinicians and promoting the shift of treatment strategies from “one-size-fits-all” to “individualized” (Table 3).

**Table 3:** Summary of representative studies on XAI in HCC treatment efficacy prediction

Treatment Type	Core Methods	Prediction Target	Clinical Significance	References
TACE Efficacy	SHAP Typing, Dynamic Trajectory Analysis	Response heterogeneity, unsuitable population, dynamic prognosis	Individualized TACE treatment, avoid ineffective treatment	[48–53]
Targeted Therapy	Multi-model Comparison + SHAP	Targeted drug response prediction, combination therapy benefit	Medication guidance, protocol optimization	[54, 55]
Immunotherapy	Pathway Analysis + Visual Interpretation of Histology (WSI), Radiomics+SHAP	Immune checkpoint inhibitor efficacy, identification of predictive histologic patterns (e.g., TILs)	Biomarker discovery, advantage population screening	[45, 47, 56, 57]
Radiotherapy	Grad-CAM Visualization, Dose Distribution Analysis	Radiation-induced liver toxicity, normal tissue complications	Treatment plan optimization, safety window definition	[25, 58]
Local Ablation	Radiomics + SHAP	Post-ablation recurrence risk, complete ablation assessment	Treatment method selection, efficacy evaluation	[59, 60]

In the prediction of Transarterial Chemoembolization (TACE) efficacy, XAI research presents a diversified and in-depth exploration. Many studies not only aim to predict efficacy but also focus on understanding the intrinsic reasons for efficacy heterogeneity. For example, Zhang et al. [48] used SHAP to subtype TACE responders, revealing efficacy heterogeneity. Furthermore, Zhang et al. [49] developed an interpretable model based on contrast-enhanced CT parameters to pre-therapeutically identify responders to conventional TACE. Wang et al. [50] focused on identifying patients unsuitable for initial TACE, providing a basis for avoiding ineffective treatment. The model of Bartnik et al. [51] revealed through XAI that the imaging features of the spleen and kidneys contributed significantly to predicting TACE outcomes, suggesting the key role of systemic inflammatory status. Li et al. [52] predicted prognosis through the dynamic trajectory of the ALBI score, introducing a time dimension. These studies collectively indicate that XAI has been shown to analyze the root causes of TACE efficacy heterogeneity, providing a preliminary scientific basis for precise patient stratification and individualized treatment strategies.

In the field of systemic therapy (including targeted therapy and immunotherapy) efficacy prediction, XAI research is moving from shallow correlation analysis to deep biological mechanism interpretation. For example, Huang et al. [54] and Ma et al. [55] quantified the contribution of clinical features to targeted therapy response. Wang et al. [35] and Lee et al. [47] associated immunotherapy efficacy with specific pathways such as cGAS-STING. Nakao et al. [56] and Chen et al. [57] searched for visual clues of immunotherapy response in baseline images. Lu et al. [53] predicted the efficacy of the TLP regimen for patients with portal vein tumor thrombus (PVTT). These explorations link macroscopic clinical presentations with microscopic biological mechanisms, helping to identify dominant beneficiary populations and reveal new biomarkers.

In addition to TACE and systemic therapy, XAI also shows application potential in the efficacy and safety prediction of other local treatment methods (such as radiotherapy and ablation therapy). For example, Liu et al. [58] and Chamseddine et al. [25] predicted radiation-induced liver toxicity. Tabari et al. [59] and An et al. [60] focused on ablation efficacy evaluation. These studies provide references for the selection of different treatment methods. These efforts provide more comprehensive decision-support information for the diversified treatment landscape of HCC, helping to balance efficacy and safety considerations.

Beyond pre-treatment radiological assessments, baseline biopsy pathology images harbor rich predictive information. As reviewed by Ding et al., AI-driven pathomics enables the quantitative extraction of high-dimensional data from whole-slide images (WSIs), offering significant potential for predicting treatment response and improving personalized therapy in HCC [45]. This approach suggests that interpretable analysis of morphological features—such as the spatial distribution and density of tumor-infiltrating lymphocytes (TILs)—from routine H&E slides could evolve into cost-effective and widely available biomarkers for therapeutic efficacy.

Despite significant progress, this field still faces a key challenge: most existing models still belong to static prediction, that is, they are good at explaining “why a patient may have poor efficacy to the current regimen” but cannot actively provide comparative decision suggestions (for example, “if the patient is switched to another treatment regimen, how might the outcome change?“). Future research needs to develop more dynamic and comparative explanatory frameworks [61, 62].

### Application of XAI in HCC prognosis prediction and postoperative management: achieving dynamic and refined risk management

In the field of prognosis prediction and postoperative management of HCC, postoperative prognosis prediction is the most concentrated direction of XAI research, and current research has formed multiple sub-directions with clear clinical orientation (Table 4).

**Table 4:** Comparative analysis of sub-directions in postoperative prognosis prediction research

Research Direction	Core Scientific Question	Characteristic Technical Method	Representative Studies	Clinical Value and Innovation Points
Clinical-Imaging Fusion	How to optimize prognosis assessment with conventional indicators?	Multiple Regression + SHAP Analysis	[63–67]	Easy for clinical promotion, strong practicality
Radiomics-Driven	How do imaging features reflect tumor biology?	Radiomics + Survival Analysis + Interpretability Mapping	[68–71]	Non-invasive prognosis assessment, discover new biomarkers
Multi-omics Integration	How to synergistically predict with multi-dimensional data?	Deep Learning Fusion + Multimodal Interpretability; Integration of pathomics with genomics/transcriptomics	[37, 39, 43, 72]	Provide mechanistic explanation, connect macro and micro; leverage histomorphology as a bridge
Special Population Customization	What is the specificity of prognosis in special subgroups?	Customized Model Development + Subgroup Analysis	[73–76]	Precise stratified management, compensate for the lack of traditional staging
Surgical Safety Assessment	How to quantify perioperative risk?	Risk Threshold Quantification + Decision Tree Analysis	[77, 78]	Surgical plan optimization, reduce complication risk
Dynamic/Long-term Prognosis	How to achieve risk assessment that changes over time?	Longitudinal Model, Trajectory Analysis	[52, 62]	More in line with the logic of chronic disease management

In prognosis prediction models based on clinical-imaging feature fusion, XAI research focuses on integrating routine clinical indicators and imaging features to build predictive tools with high clinical practicality. For example, Zhang et al. [63] developed a prognostic nomogram based on CECT radiomics. Liu et al. [64] and Yu et al. [65] focused on postoperative recurrence prediction of HBV-related HCC. Wang et al. [66] and Li et al. [79] focused on the prognosis of large hepatocellular carcinoma. This type of research builds practical prognostic prediction tools that are easy to clinically promote by integrating easily accessible clinical and imaging data.

At the level of deep prognostic mechanism exploration driven by radiomics, XAI research focuses on revealing the intrinsic relationship between imaging features and tumor biological behavior. For example, Zhang et al. [68] used random survival forests to find that MVI status is a key predictive factor. Qin et al. [69] first applied MRI habitat imaging to prognosis prediction. Wei et al. [70] focused on dynamic changes in liver function during the perioperative period. The significance of these studies is to non-invasively assess the biological behavior of tumors, such as invasiveness, through radiomic features, providing a deeper explanation for prognosis judgment.

In the direction of multi-omics integrated prognosis prediction systems, XAI research is committed to integrating multi-dimensional data such as genome, transcriptome, and imaging. For example, Gao et al. [43] integrated single-cell transcriptomics and radiomics. Peng et al. [72] developed a multimodal fusion model based on Grad-CAM. The advantage of this type of research is that it can provide a potential biological explanation for prognostic differences from a system level, promoting prognosis prediction towards precision medicine.

In precise prognosis prediction for special clinical scenarios, XAI research focuses on specific clinical subgroups, reflecting the potential for precise application. For example, Li et al. [73] developed a prediction model for AFP-negative patients, Wu et al. [74] focused on the population with diabetes complicated by HCC, Yang et al. [75] and Zhang et al. [71] focused on the prediction of large hepatocellular carcinoma and early recurrence risk. Chen et al. [76] focused on the risk prediction of spontaneous rupture. These studies targeting special populations demonstrate the application value of XAI in achieving more individualized prognosis assessment.

In the field of surgical complications and perioperative risk assessment, XAI research focuses on risk prediction related to surgical safety. For example, Tang et al. [77] and Zhong et al. [78] assessed the risk of liver failure after hepatectomy. Altaf et al. focused on complications such as bile leakage [67]. These studies provide important decision support for surgeons to formulate perioperative management strategies and surgical plans.

In dynamic and long-term prognosis assessment, XAI research introduces a time dimension, achieving dynamic assessment of prognosis. For example, Li et al. [52] predicted prognosis through the trajectory of the ALBI score. Nakahara et al. [62] constructed a risk prediction model for HCC after HCV cure. These dynamic assessment studies are more in line with the long-term management needs of HCC as a chronic disease, making prognosis prediction more clinically practical.

Digital pathology images also serve as a foundation for building interpretable prognostic models. The prognostic value of pathomic features is often elucidated through XAI. For instance, a two-stage Cox-nnet model first extracts deep features from WSIs and then fuses them with transcriptomic data for survival analysis, using interpretability methods to identify the most predictive morphological and molecular feature sets [39]. This demonstrates how XAI can help establish and explain links between morphological phenotypes on WSIs and patient outcomes, providing a novel, non-invasive layer for risk stratification that complements clinical and radiological models.

Despite notable research advances, this field continues to grapple with several critical challenges. The majority of existing predictive models are built upon historical data, making them inherently static. They lack capabilities for continuous learning and self-updating, thereby failing to keep pace with the rapidly evolving clinical landscape—such as the emergence of novel treatment regimens and updated clinical guidelines. Key limitations include model rigidity, which hinders the integration of emerging treatment paradigms, and the absence of an effective link between predictions and specific clinical interventions. Addressing these challenges is pivotal to advancing the genuine clinical translation of XAI in the realm of prognostic prediction [28, 46].

Collectively, the XAI applications reviewed in this section converge on a unified analytical pipeline—from multi-modal data inputs through interpretable processing to clinically actionable predictions—as summarized in Fig. 2.

## The path to a breakthrough: next-generation XAI technical pathways to address current challenges

Although XAI has made significant progress across various domains of HCC, its development has been constrained by the “post-hoc explanation” paradigm, exemplified by methods like SHAP and Grad-CAM. This paradigm faces three inherent bottlenecks: correlation rather than causation, the semantic gap, and static prediction [80–82]. To overcome these limitations, a fundamental paradigm shift is necessary – moving from “explaining black-box models” to “building intrinsically interpretable models,” and deeply integrating explanations with dynamic clinical decision-making processes.

### From “statistical correlation” to “causal inference”: providing reliable evidence for biomarkers and treatment decisions

In current HCC biomarker research, correlation-based analysis methods dominate, but this approach struggles to distinguish genuine causal drivers from mere epiphenomena. Causal machine learning methods, such as double machine learning and causal forests, offer new possibilities to break through this limitation [83, 84]. These methods can more accurately estimate the causal effect between variables by controlling for known confounders like age, liver function, and treatment history. Specifically, integrating a causal inference framework with multi-omics integration models [43] can transform the questions asked in biomarker research. Investigations can move beyond “which gene expressions are associated with prognosis” to further probe “to what extent intervening on a specific gene expression can alter patient prognosis” [34]. Similarly, in treatment decision support, causal inference methods can enhance existing efficacy prediction models [54], enabling them not only to predict the probability of a patient’s response to a specific treatment regimen but also to estimate the potential benefit of switching to an alternative regimen through counterfactual reasoning. The application of this approach may provide a more reliable evidence base for precision medicine in HCC.

To operationalize this paradigm shift from correlation to causation, several advanced causal inference methodologies are pivotal. Double machine learning (DML) employs a two-stage estimation process to robustly control for high-dimensional confounders, enabling the estimation of the average treatment effect from observational data. For instance, DML has been used to quantify the causal effect of modifiable intraoperative variables on surgical outcomes [85]. Counterfactual reasoning answers clinically actionable “what-if” questions by modeling potential outcomes under different interventions. In HCC, this approach can assess the survival benefit of alternative treatment strategies that deviate from standard guidelines, providing a quantitative basis for personalized decision-making [86]. Causal forests, an extension of random forests, are specifically designed to estimate heterogeneous treatment effects. They can identify patient subgroups that benefit differentially from a given therapy. A seminal application in HCC used causal forest to reveal that patients with tumors ≤ 5 cm and non-smooth margins derive significant survival benefit from anatomical resection, whereas others do not, directly enabling precision surgical planning [87]. Together, these methods provide a potential technical foundation for building robust, explainable, and actionable causal models in HCC research.

### From “pixel heatmaps” to “concept-driven”: bridging the semantic gap in image analysis

A significant semantic gap—the discrepancy between pixel-level heatmap highlights and the high-level clinical concepts that radiologists use for diagnosis—exists between current heatmap-based explanation methods and the diagnostic reasoning of clinicians. Concept Bottleneck Models (CBM), a class of inherently interpretable models that first predict human-defined clinical concepts and then use these concepts to make the final decision — previewed in the multi-omics pipeline (Fig. 2) — offer a new direction to address this issue [88]. This model requires the network’s intermediate layers to learn human-understandable high-level semantic concepts, thereby establishing a direct link between the model’s decision process and clinical cognition. In medical imaging, CBMs can be integrated with the LI-RADS classification system [9, 10]. By explicitly incorporating clinical concepts like “nonperipheral washout in the arterial phase” or “washout appearance” into the model, the AI system’s decision-making process can be aligned more closely with radiologists’ diagnostic logic. In pathological image analysis, this model can integrate quantitative morphological indicators like “nuclear atypia” and “chromatin density” [22], grounding the model’s decisions in morphological features recognized by pathologists. The application of this approach has the potential to significantly enhance the acceptance and trustworthiness of AI systems in clinical practice.

The “concept-driven” paradigm must also evolve to address the emerging challenge of interpreting large-scale pathology foundation models (e.g., PLUTO), which are pre-trained on vast corpora of WSIs to produce powerful, general-purpose image embeddings. Understanding these foundational “black boxes” requires shifting from explaining task-specific outputs to mechanistically interpreting their internal representations—a frontier known as mechanistic interpretability. Pioneering work in this direction has applied Sparse Autoencoders (SAEs), a technique designed to learn sparse, overcomplete representations, to the embeddings of a pathology foundation model [89]. The study demonstrated that individual SAE latent dimensions (neurons) could be strongly and monosemantically correlated with specific biological concepts, such as “plasma cell count” or “lymphocyte count,” and were robust to technical confounders like scanner and stain variation. This suggests that techniques like SAEs can begin to “disentangle” the complex, polysemantic representations of foundation models into human-understandable, elemental biological concepts. This research direction marks a significant evolution: from providing post-hoc explanations for a model’s decision towards anatomizing the internal semantic structure of the model itself, offering a potential path to truly transparent and trustworthy general-purpose medical AI.

### From “static snapshots” to “dynamic narratives”: incorporating continual learning and counterfactual explanations

Most current prognostic prediction models are built on static data, making them ill-suited to the dynamic, evolving nature of clinical practice data. Continual Learning algorithms provide a technical foundation to address this issue [90]. These algorithms enable models to learn continuously from new clinical data without catastrophically forgetting previously acquired knowledge, thereby facilitating dynamic model updates. In prognostic prediction, continual learning can be combined with dynamic trajectory analysis [52]. By continuously integrating imaging, laboratory tests, and new treatment information from patient follow-ups, the model has the potential to achieve real-time, dynamic risk assessment for individualized prognosis. Furthermore, incorporating a counterfactual explanation framework [91] can enhance the model’s clinical utility. For instance, for a patient predicted to be at high risk of recurrence, the model could not only provide the risk prediction but also generate specific, actionable suggestions, proposing that “optimizing a key liver function parameter (e.g., the ALBI score) might reduce the estimated recurrence risk”. This dynamic and actionable prediction model better aligns with the clinical needs of managing HCC as a chronic condition.

### From “model-centric” to “human-AI collaboration”: building interactive explainable systems

Current XAI systems mostly provide one-way information output, lacking deep interactive capabilities with clinical experts. Interactive systems based on counterfactual questioning and case-base retrieval [5] have the potential to change this situation, promoting the evolution of XAI from an “explanation tool” to a “decision partner.” In a practical clinical scenario, when a model flags a patient as high-risk based on features like “peritumoral textural entropy,” the physician could use an interactive interface to pose a query: “Show me cases with similar features that had a good prognosis” [43]. By retrieving a database of similar cases, the system might reveal underlying distinguishing factors, such as a “specific immune cell infiltration pattern.” This human-AI collaborative decision-making mode not only enhances the clinician’s trust in the AI’s suggestions but can also foster the generation of new scientific hypotheses.

## Discussion and conclusions

### Summary of key findings

This review identifies several cross-cutting insights that emerge when XAI applications across HCC imaging, biomarker discovery, treatment prediction, and prognosis assessment are examined collectively. First, the predominant XAI paradigm remains post-hoc explanation—methods such as SHAP and Grad-CAM are widely adopted across all four domains, yet their inherent limitations (correlational reasoning, semantic gaps, and static outputs) are equally pervasive. Second, XAI has enabled a progressive deepening of biological insight, from identifying isolated biomarkers to mapping functional gene modules and pathways, and further to integrating multi-omics data with imaging phenotypes—a trajectory that reflects the field’s movement toward system-level understanding. Third, a consistent gap exists between explanation and action: most current models explain why a prediction was made but cannot yet inform what clinical intervention should follow. As noted in the Introduction, the pronounced heterogeneity of HCC amplifies the need for XAI; by revealing the features driving individual predictions, XAI can help distinguish between patient subgroups that may respond differently to the same intervention, addressing a need that conventional black-box models cannot meet.

These observations collectively underscore a central tension in current XAI research for HCC: while XAI has succeeded in making AI predictions more transparent, it has not yet made them more actionable. Bridging this gap—from explanation to decision support—represents the next frontier.

### Limitations of this review

Several limitations of this review should be acknowledged. First, this is a narrative synthesis rather than a systematic review with a predefined protocol; while we aimed to cover the major application domains of XAI in HCC, the literature selection may be subject to author bias and relevant studies may have been inadvertently omitted. Second, this review does not include a quantitative meta-analysis. The decision was guided by the considerable methodological heterogeneity among current XAI studies, which vary widely in model architectures, evaluation metrics, and clinical endpoints. While some radiomics-based studies have employed meta-analytic pooling of diagnostic performance metrics [46], the XAI field has not yet adopted standardized quantitative benchmarks that would allow meaningful meta-analytic aggregation. As the field matures and reporting standards for XAI evaluation become established, future systematic reviews incorporating meta-analytic components will be valuable for quantitatively benchmarking the added value of different explainability techniques. Third, the reviewed studies span different imaging modalities, molecular data types, and clinical endpoints, making direct comparison across studies challenging. Finally, the rapid pace of XAI development means that newer methods may have emerged after our literature search was completed.

### Paradigm shift and future directions

A systematic analysis of existing research demonstrates that XAI has shown significant value across multiple key areas of HCC research. From achieving semantic alignment between AI decisions and LI-RADS standards in imaging diagnosis, to identifying functionally significant modules in biomarker discovery; from revealing the drivers of heterogeneous treatment responses in therapeutic efficacy prediction, to enabling dynamic risk stratification in prognosis assessment—these achievements collectively outline a clear picture of how XAI enhances clinical insight. However, when these advancements are examined against the practical demands of clinical decision-making, the limitations of the current post-hoc explanation paradigm, represented by techniques such as SHAP and Grad-CAM, become apparent. This “black-box explanation” paradigm inherently suffers from three core limitations: its explanations remain at the level of statistical correlation, lacking causal support; its visualization results (e.g., heatmaps) create a semantic gap with the conceptual thinking of clinicians; and its static, passive explanation mode struggles to adapt to the dynamically evolving clinical environment.

These limitations highlight the need for a potential paradigm shift in XAI research for HCC. The core of future development lies in shifting from “explaining trained black-box models” to “constructing intrinsically understandable model systems,” and deeply coupling the explanation process with clinical decision-making workflows. This transformation unfolds along three interconnected dimensions:

First, on the methodological level, the focus should advance from inferring correlations to establishing causal inference. Integrating causal reasoning frameworks like double machine learning and causal forests into XAI can not only distinguish causal drivers from incidental associations but also provide stronger evidential support for biomarker validation and comparative effectiveness research of treatment regimens. For instance, when evaluating targeted therapy efficacy, a model should be able to answer “what benefit would result from switching to an alternative targeted drug?” rather than merely stating “the probability that the current drug is effective.”

Second, on the model design level, the semantic gap between low-level features and high-level clinical concepts needs to be bridged. Intrinsically interpretable architectures like Concept Bottleneck Models (CBM) offer a viable path. By explicitly embedding clinically familiar concepts (e.g., “arterial phase rapid washout,” “microvascular invasion on histology”) into the model’s decision process, AI reasoning can be naturally aligned with clinical cognition. This concept-driven approach appears particularly suitable for radiomics and pathological image analysis, as it directs the model’s attention to structural features with clear clinical relevance.

Finally, on the system level, XAI needs to evolve from a static tool into a dynamic clinical collaborative partner. This requires integrating two key technologies: ⑴ Counterfactual explanation generation, which provides clinicians with actionable insights such as “how would the outcome change if a different intervention were taken?“. ⑵ Continual learning capabilities, enabling models to adapt to the evolution of disease management strategies by continuously learning from new clinical data streams without forgetting previously acquired knowledge. More importantly, next-generation XAI systems should be interactive, supporting clinicians in questioning, exploring, and comparing explanations based on cases, which can achieve genuine human-machine collaboration in complex decision-making scenarios.

Furthermore, it is crucial to note that translating these technical blueprints into widespread clinical practice encounters significant accessibility barriers. These barriers stem from multiple practical obstacles: first, the pervasive lack of digital infrastructure, particularly in pathology where whole-slide imaging is far from ubiquitous, which is a prerequisite for data-intensive paradigms like continual learning [21, 45]; moreover, the adoption of advanced XAI tools even in more digitally advanced fields like medical imaging has been observed to lag behind other industries [92, 93]. Second, the high costs and technical complexity of deploying, maintaining, and updating advanced XAI systems hinder their widespread adoption in most clinical centers, a concern underscored by health economic analyses that identify cost as a key determinant of viability [94]. Finally, the significant effort required to seamlessly integrate these tools into heterogeneous clinical workflows and adapt them to varying levels of user expertise constitutes a major implementation challenge, a barrier frequently cited across both pathology and radiology applications [95]. Addressing these barriers requires concerted, parallel efforts in health technology policy, resource allocation, and systems engineering, alongside core algorithmic innovation.

The realization of this vision faces numerous challenges, including the construction of high-quality multimodal datasets, the research design required for causal inference, and the prospective validation of XAI’s clinical utility. However, as explainability technologies become more deeply integrated with clinical practice, XAI is poised to evolve beyond a tool for explaining model decisions. It may evolve into a tool that enhances clinical cognition and supports precise decision-making, thereby advancing HCC diagnosis and treatment towards higher levels of individualization and precision.

### Conclusions

XAI has enabled substantial progress in translating black-box AI predictions into clinically interpretable insights across the HCC research landscape—from imaging diagnosis and biomarker discovery to treatment efficacy prediction and prognosis assessment. However, the field remains at a transitional stage: current post-hoc explanation methods, while widely adopted, have not yet bridged the gap between making predictions transparent and making them clinically actionable. Moving from statistical correlation to causal reasoning, from pixel-level heatmaps to concept-driven explanations, and from static models to dynamic, interactive clinical partners represents the central challenges for the next generation of XAI in HCC. Addressing these challenges will determine whether XAI fulfills its potential as a decision-making partner in HCC precision medicine.

## Data Availability

No datasets were generated or analysed during the current study.
